# Supplementary material for: Are carbon emissions trading and green financial instruments synergistic? -Comprehensive quantitative research based on content analysis
Source: PLoS One. 2024 Mar 7;19(3):e0298601. doi: 10.1371/journal.pone.0298601 (PMC10919601; doi:10.1371/journal.pone.0298601)
Supplement: S1 Data — (ZIP) [file pone.0298601.s001.zip › Data/地级市Z_F工作报告.zip!/┴╔─■/▒╛╧¬╩╨/└┤╫╘╣1⁄2╓┌║┼í╛┬φ┐╦╩2╛▌═°í┐_2013.docx]

各位代表：

　　现在，我代表市人民政府向大会报告工作，请予审议，并请市政协委员和各位列席同志提出意见和建议。

　　一、2012年工作总结和五年工作简要回顾

　　2012年是本届政府任期的最后一年，经过全市上下的共同努力，完成了各项年度任务。

　　（一）坚持稳中求进、稳中求快，实现了各项主要指标增幅均高于全省平均水平。预计，全市生产总值完成1130亿元，同比增长13%；公共财政预算收入完成122.3亿元，增长19.4%；固定资产投资完成743亿元，增长28%；规模以上工业增加值完成560亿元，增长15%；社会消费品零售总额实现263亿元，增长16%；实际利用外资4.6亿美元，增长31.4%；出口创汇23亿美元，增长18.6%；引进省外投资到位资金280亿元，增长51%；城镇居民人均可支配收入22518元，增长14%；农民人均纯收入11048元，增长16%。

　　（二）全力以赴争政策、抢项目、抓落实，项目年活动成效显著。采取市级领导包扶、月通报、季调度等措施，全力推进了1138个项目建设，全年竣工项目504个。一批能源交通重大项目取得新进展，投资30亿元的本溪热电联产项目获得“路条”批复；总投资26.6亿元的田桓铁路初步设计获得批复；总投资108亿元的沈丹客专工程进展顺利；建成钢铁大道、卧龙大桥；500千伏程家变电站等建成运营；丹阜高速石桥子互通立交改造开工建设。坚持对外开放，全年组织国内外重要招商活动30次，引进境外和域外投资项目663个。积极向上争取政策和资金，溪湖区、南芬区国家级资源综合利用示范基地正式获批，高新区18亿元城投债券获批发行；全市共争取省级以上专项资金52亿元，增长30.9%。

　　（三）全面开展政企合作，钢都建设扎实推进。认真落实市政府与本钢集团签署的战略框架协议，全力支持本钢集团发展，服务本钢集团的15件实事已完成9件。依托本钢集团拉长钢材深加工产业链，全市钢材深加工产业园区新入驻项目44个、开工建设项目37个、竣工投产项目47个，实现销售收入176亿元。本钢集团积极启动冷轧高强钢改造等重点项目建设，调结构、降成本、强化企业管理，保持了生产经营的平稳运行，为全市经济平稳较快增长做出了决定性贡献。

　　（四）实施“181”发展战略，药都建设实现新突破。设计了“181”的发展载体和框架格局，实施五个“百日决战”，全方位推进中国药都建设。坚持产业为重，全年新开工产业项目11个，新投产药业企业8户，引进产业项目18个，储备产业项目30个，药业及相关配套产业实现销售收入200亿元。坚持科技支撑，德国拜耳、华大基因等一批高层次科研团队、研发项目入驻药都，依托创新园建设的8个省级公共技术服务平台全面投入运营。坚持大学为要，沈阳药科大学南校区招生办学，中国医科大学盛京医院医药研发教育基地开工建设，沈阳药科大学主校区、辽宁中医药大学本溪校区完成规划设计，辽宁医药化工职业技术学院10栋单体建筑主体封顶。同时，市高中北校区投入使用，省实验学校本溪分校、榆林华苑幼儿园启动建设。坚持产城结合，完成道路、绿化等基础设施投资16.5亿元，会展中心、22万平方米标准化厂房主体封顶，易辰药都商贸城和绿地中央文化广场启动建设。新开通了3条区内公交线路。在高新区率先共享024区号。成功举办了第四届药交会和2012’海峡两岸医药卫生交流与合作会议。本溪高新区成功升级为国家级高新区，成为创新型国家建设的重要组成部分。

　　（五）发展温泉旅游度假产业，枫叶之都建设全面铺开。实施“5-10-20”温泉旅游发展战略，水洞温泉度假区重点项目征地动迁工作进展顺利，希尔斯、天汤等20个温泉项目开工建设。本溪水洞、关门山、老边沟等重点景区基础设施不断完善。“十一”黄金周等节假日期间旅游市场持续火爆，以枫叶为代表的山水生态游全线飘红，全年接待国内外游客分别增长40%和56.9%。我市被授予“中国温泉之城”称号，又增添了一张亮丽的城市名片。

　　（六）狠抓工业“五项工程”，工业经济保持平稳增长。从政策、环境、服务等多方面入手，统筹和配置各项生产要素，千方百计组织好企业运行，全市工业经济实现企稳回升，工业“五项工程”取得新进展。全年实施投资千万元以上工业项目418个，完成工业固定资产投资400亿元；实施企业并购项目4个；9个产业集群实现销售收入630亿元，增长41%；万元增加值能耗下降率6%，淘汰5户企业落后产能。当年新增规模以上工业企业100户，全市总量达到537户，向进军千户规上企业目标迈出了重要一步。

　　（七）积极推进“三化”进程，县域经济发展驶入快车道。结合“一县一业”发展现代农业，桓仁县新增酿酒葡萄示范区1万亩，获批“中国山参之乡”；本溪县新增地产中药材1.02万亩，获批“中国刺五加之乡”，“本溪辽五味”通过国家GAP认证。两县工业产业集群入驻项目92个，其中玉晶玻璃一期、沈煤龙盟新型环保材料等龙头项目竣工投产。两县房地产开工240万平方米，竣工123万平方米。在全省44个县的县域经济综合评价中，本溪县位列综合实力序列第15位，桓仁县位列进步最快序列第10位。

　　（八）加快发展房地产等服务业，城区经济稳步发展。全年房地产新开工700万平方米，竣工400万平方米；商品房销售面积460万平方米，销售额169亿元，实现了双增长15%目标。商贸流通业不断壮大，构建了物流产业“6园区、1中心”的发展格局。强化市场监管监测，城乡市场繁荣兴旺。城区服务业集聚区建设稳步推进，平山中央商贸商务集聚区金融大厦等项目开工建设，明山现代服务业集聚区通过省级认定，彩北商贸物流中心主体工程封顶，南芬区建成辽砚会展中心。平山区、明山区在全省56个城区综合考评中，分列第8位和第15位。

　　（九）实施“青山、碧水、蓝天工程”及“创模、创卫”工作，城乡环境面貌不断改善。青山工程在“三区两带一企”实施治理面积3万余亩，超额完成省定年度指标。碧水工程完成本钢集团污水“零排放”等项目24个，国控老官砬子断面达到二类水质。蓝天工程完成大气治理项目27个，减排二氧化硫2600吨、氮氧化物2038吨。实施了北沙河、太子河、浑江河道综合整治工程。新建日供水10.5万吨的桥北和李家山两座水厂。按照“创模”、“创卫”要求，开展了市容市貌专项整治活动。建成20处景观带，绿化栽植树木86.8万株，新建、改造绿地79.2万平方米。开工建设了北地跨线桥一期工程，改造了峪明路、东明路等9条城市道路，拆除了东芬转盘和铁路转盘，提升了主城区主干路网功能。投资6500万元，更新180辆公交车上线运营。

　　（十）努力推动改革创新，有效破解了一批要素瓶颈问题。市政府带领47个市直、中省直部门先行搬迁至高新区，与高新区合署办公，并在高新区构建了“1+4”的工作推进体系。继续深化扩权强县区，五女山、太子河和桥北三个市级经济开发区升格为省级。通过银行贷款、表外业务、发行城投债券等方式，全市融资总额310亿元，银行业金融机构贷款余额710亿元。首期规模9.8亿元的三支基金正式入驻药都。多渠道、多方式争取用地指标，全年共用地1531公顷，保障了重点项目建设空间。

　　（十一）全面发展各项社会事业，积极构建和谐社会。启动“基础教育强县区”工程，完成37所幼儿园、城区相对薄弱学校以及农村标准化寄宿学校的改造建设任务。成功举办了市第十三届文化艺术节暨首届群众文化节。剪纸文化创意等文化产业园建设进展顺利。“十二运”筹备工作稳步推进。医药卫生体制改革三年五项重点任务全面完成。人口和计划生育综合改革不断深化。市爱卫会被评为全国爱国卫生工作先进集体。食品药品监管工作进一步加强。无规定动物疫病区建设通过国家验收。新组建了市邮政管理局。一批信访疑难问题和信访积案得到有效化解。排查治理各种隐患8795项，全市安全生产形势总体稳定。以“十八大”安保为主线，加强公共安全管理，强力推进了城乡警务改革。外事、侨务、对台、残联、民族、宗教、人防、档案、方志、地震、气象等工作也取得新的成绩。

　　（十二）落实十二件民生实事，切实保障和改善民生。一是完成保障性住房6307套，新建城市、国有工矿、中央下放煤矿和垦区棚改房20782套。二是实现城镇实名制就业7.7万人。三是企业退休人员养老金月人均增加200元，失业金月人均增加110元，城市低保月人均增加60元，农村低保年人均增加600元。四是将2474名原“五七工”、“家属工”纳入养老保险统筹体系。五是基本解决了5.2万名国有和集体困难企业在职职工基本医疗保险问题。六是将全市1.5万名老工伤人员和3.5万名事业单位人员纳入了工伤保险统筹。七是144所中小学校全部实现义务教育“班班通”。八是实施国家基本药物制度，覆盖人口140万。九是投资6.3亿元，实施“暖房子”工程60.5万平方米，改造热源10处、管网120公里，供热管网成新率提高到66%，450万平方米低温区得到改善，受益居民10万多户。十是维修了62个住宅区的人行甬路和车行路，绿化了32个住宅区。改造整治街巷路、“摸黑路”123条。十一是建设了48处农村饮水安全工程。十二是新增菜田7319亩。

　　（十三）执政为民、重在落实，政府自身建设进一步加强。市政府系统实施了“四位一体”的工作推进模式，并将重点工作设计出任务书、时间表、路线图加以落实，沈丹客专、本钢冷轧高强钢和地工路项目征地拆迁等难点问题有效解决。办理人大代表建议和政协提案874件，办复率100%，解决率70.5%。制定地方性法规2件，制定和修改政府规章6件，办理行政复议案件150件。受理办结各类审批事项23.5万件，办结率99.7%。落实政府系统党风廉政建设责任制，加强行政监察和审计监督，市公共行政服务中心和市公共资源交易中心新址投入使用。政府督察工作办结率和回复率达到98.6%和98.2%。

　　各位代表，2008年以来这五年，是我市经济实力明显增强，产业结构调整重大突破的五年；是沈本一体化迈出坚实步伐，“三都五城”建设加快推进的五年；是基础设施不断完善，城市功能和形象显著提升的五年；是社会建设不断进步，人民群众得到更多实惠的五年。突出表现在以下七个方面：

　　——综合实力明显提升。五年中，全市生产总值增长1.5倍，公共财政预算收入增长2.9倍，固定资产投资增长3.8倍，规模以上工业增加值增长1.5倍，社会消费品零售总额增长1.3倍。五年中，全市上下还创造了宝贵的精神财富。“昂首十二五、建设新本溪”成为全市人民奋进的目标；建设“三都五城”历程中形成的本溪精神，正在成为团结全市人民拼搏进取的强大精神动力。

　　——产业格局多元突破。依托本钢集团打造钢都，成功实现本钢、北钢合并重组，规划建设了桥北、东风湖等钢材深加工产业园，推进了本钢冷轧高强钢、思山岭铁矿等重大项目。依托高新区打造药都，累计引入生物医药产业及科研项目138个，85家企业和科研机构投产运营。以建设水洞温泉旅游度假区为切入点，加快发展旅游度假产业，我市被授予“中国枫叶之都”称号。实施工业“五项工程”，全市形成了9个工业产业集群。中药材、山野菜、食用菌等现代农业特色产业已形成规模化、产业化。房地产市场步入提档升级阶段，上海绿地和恒大、佳兆业等知名房地产企业进入本溪。商业网点布局更加合理，全市商品交易市场达到135个。县域经济和城区经济分别完成首轮倍增和翻番目标，成为全市发展的重要支撑力量。

　　——城乡建设加快推进。主城区向东拓展13平方公里，新建、改造和整治了37条主次干路，全面告别了“摸黑路”。沈本新城初具规模，建成区面积超过20平方公里，集聚人口10万人。沈本产业大道、桓仁“两高一铁”等一批重大交通项目投入使用，全市已形成“一横、七纵、三环”的公路交通网络。新修加固堤防531公里，新建改造城市净水厂4座、农村饮水安全工程279处，防洪减灾和供水保障能力不断提高。全市森林覆盖率达76%，位居全国前列。新增绿地569.8万平方米，人均公共绿地9.95平方米。实施重点污染源治理100多项，城市环境综合整治定量考核排名升至全省第4位。五年间，我市成功创建了国家森林城市、国家园林城市和省卫生城市、省环保模范城市。本溪正在由一个“卫星看不见”的城市，建设成一个“让人看不够”的城市。

　　——改革开放不断深化。集体林产权制度改革成为全国的一面旗帜。扩大了县区管理权限，创新了高新区和各产业园区的体制机制。调整完善了市县（区）的财政管理体制。非公经济实现增加值占全市GDP比重提高到60%。大力发展外向型经济，五年累计实际利用外资14.2亿美元，引进省外投资到位资金800亿元，出口创汇88.9亿美元。本钢集团在国内钢铁行业中连续三年实现出口总量第一。

　　——社会事业全面进步。教育事业形成了从学前教育、基础教育到职业教育结构合理、科学有序发展的良好局面。依托高新区引入医药类相关高校6所，本溪拥有高校数量跃居全省第三位。深化医药卫生体制改革成效显著，覆盖城乡居民的基本医疗卫生制度初步建立。公共文化服务基本实现了乡镇、社区和行政村文化活动场所的全覆盖。广播电视村村通、有线电视数字化全面完成。文化产业已搭建起“一县一园”、“一区一品”的发展架构。五年间，我市还先后荣获民族团结进步模范市“五连冠”、双拥模范城“七连冠”等荣誉称号。

　　——人民生活持续改善。五年来，全市城乡居民人均收入实现翻倍增长。居民消费价格平均上涨2.9%，为全省市场价格最平稳的城市之一。完成采煤沉陷区治理收尾工作，城市集中连片和国有工矿棚户区改造等安置居民4万余户。累计实名制就业39.2万人。企业退休人员养老金翻了一番，基本养老和医疗保障实现城乡全覆盖，城乡低保实现动态化应保尽保，各项民生保障标准均提高到全省平均水平以上。及时回应和妥善解决了大量信访问题。安全生产监管和整治力度进一步加大。

　　——自身建设不断加强。自觉接受人大、政协法律监督和民主监督，五年办理人大代表建议和政协提案4954件，办结率100%，解决率70.8%。制定地方性法规草案13件、政府规章31件，办理行政复议案件640件。市级行政审批项目精简90.8%，保留126项。全面深化和拓展“五大系统”建设，对10.9万件审批事项实施了电子监察。加强政风行风建设，“行风热线”和“民心网”受理、答复和解决群众诉求9230件，满意率达95%以上。绩效考核评价机制不断加强，市政府绩效考评工作连续两年位居全省第三位。

　　各位代表，过去五年的成果来之不易，这是省委、省政府和市委的正确领导、市人大和市政协的监督支持、历任领导打下的坚实基础、全市人民团结奋斗的结果。在此，我代表市政府，向全市各族人民，向驻溪部队、武警官兵和公安干警，向各民主党派、工商联、人民团体、无党派人士，向关心、支持和参与本溪发展建设的各界朋友，表示衷心的感谢和崇高的敬意!

　　回顾过去五年工作，我们深刻体会到：一是必须坚持解放思想、改革开放，从本溪实际出发，跳出本溪看本溪，以思想解放和改革开放来推动各项工作加快发展；二是必须坚持一心一意谋发展，心无旁骛抓落实，按照市第十一次党代会及“十二五”规划确定的目标方向，一张蓝图绘到底；三是必须坚持为人民服务宗旨，将改革发展成果不断惠及广大人民群众；四是必须坚持以本溪精神凝聚力量，在全市营造政通人和、共谋发展的良好氛围。

　　回顾本届政府的工作，我们也清醒认识到存在的差距和面临的问题：全市经济结构调整任务艰巨，产业集群规模不大，服务业发展急需加快，城乡居民增收能力有待增强，解决民生问题任务繁重，政府工作效率还需提高等等。

　　二、今后五年政府工作的总体要求和重点任务

　　未来五年，是我市深入贯彻落实党的十八大精神、全面建成小康社会的关键时期。我们既面临着经济全球化和国内先进地区产业转移、沈阳经济区战略加快实施等诸多机遇，也面临着宏观环境复杂多变、城市发展竞争激烈的严峻挑战。我们必须始终保持清醒头脑，切实增强紧迫感和责任感，牢牢把握机遇，主动应对挑战，加倍努力工作，不断开创经济社会发展的新局面。

　　建议新一届政府工作的总体要求和奋斗目标是：深入贯彻党的十八大和省、市第十一次党代会精神，以科学发展为主题，以转变经济发展方式为主线，坚持开放引领，强化创新驱动，壮大产业支撑，突出民生优先，着力推进本溪的新型工业化、信息化、城镇化、农业现代化和市场化、国际化进程，加快沈本一体化和“三都五城”建设步伐，加强经济、政治、文化、社会和生态文明建设，全力建设富庶文明幸福新本溪。未来五年，全市各项主要经济指标力争实现两位数增长。到2017年，全市经济总量和公共财政预算收入力争翻一番，率先全面建成小康社会。

　　新一届政府应重点抓好以下六项任务：

　　——产业结构调整优化升级。进一步加快生物医药、钢材深加工、旅游度假三大产业集群建设，把本溪建设成全国一流的生物医药产业基地、精品钢材加工产业基地和“双世界遗产”旅游产业强市。落实“1+9”工业发展布局，支持本钢集团进入世界500强，推动9大产业集群尽快形成百亿元产业规模。大力发展信息技术、新能源、新材料等战略性新兴产业和房地产、物流经济、总部经济、会展经济等现代服务业，形成多元产业支撑的新格局。

　　——城市空间布局实现突破。拓展城市发展空间，形成“本溪主城、沈本新城、观音阁新城、桓仁新城”四大城市区域。完成市级行政中心向沈本新城迁移任务，基本实现沈本一体化。坚持产业带和城市带有机互动、同步发展，加快城镇化步伐，构建主城、新城、新市镇网络格局，形成布局合理、环境优美、特色鲜明的城市带。到2017年，全市城镇化率达到80%以上。

　　——改革开放创新继续深化。推进农业、农村综合配套改革和文化、医药卫生等社会事业改革。深化行政审批制度改革和事业单位分类改革。创新产业园区体制机制。完善财政管理体制，加强融资平台管理，积极推进企业上市。继续深化国企改革，支持非公经济加快发展。形成全方位开放型经济体系新格局。以高新区为龙头，强力推进创新型城市建设，创业创新创优工作走在全省前列。

　　——生态文明建设不断加强。完成国家环保模范城市、国家卫生城市创建任务，积极创建国家生态市，做美丽中国的典范城市。到2017年，全市有林地面积达到960万亩，森林蓄积量6000万立方米，森林覆盖率稳定在76%以上，继续保持全国领先、全省首位。累计新建改造绿地350公顷，绿化覆盖率达到40%以上。累计治理水土流失面积40万亩，河道生态治理5万亩。全市单位生产总值综合能耗年均下降3.7%。继续保持城市环境综合整治定量考核排名全省前4位。

　　——和谐社会发展全面推进。形成较为完善的教育、文化、体育、医疗卫生和人口计生等公共服务体系。倡导本溪精神。加快建设文化强市。争创全国文明城市。加强安全生产管理，形成规范的安全生产法治秩序。形成听取群众意见、回应群众诉求、解决群众问题的信访工作机制。建设“平安本溪”，加强公共安全管理。社会建设工作走在全省前列。

　　——人民生活水平稳步提高。完成覆盖城乡所有居民的社保体系建设任务，健全社会救助和保障标准正常调整机制；保障低收入困难家庭廉租住房，消除城区棚户区；民生保障水平进入全省前列。基本实现城乡充分就业，未来五年新增就业30万人，城镇登记失业率控制在4.5%以内。继续保持物价稳定，确保城市居民消费价格增幅低于全省水平。千方百计提高城乡居民收入水平，实现城乡居民收入高于经济增长速度、高于全省平均水平。

　　三、2013年的主要任务

　　今年工作的总体要求是：落实市委十一届四次全会精神，坚持科学发展，坚持转变经济发展方式，坚持稳中求进，实现“首季开门红、半年双过半、全年重点任务提前完”。

　　各项主要指标计划安排是：全市生产总值增长10%；公共财政预算收入增长15%；规模以上工业增加值增长11%；固定资产投资增长20%；社会消费品零售总额增长12%；实际利用外资增长20%；出口创汇增长15%；引进省外投资到位资金增长20%；城乡居民人均收入增长10.5%。今年要重点抓好以下工作：

　　第一，扎实推进改革开放，为加快发展增添新活力。坚持改革不停顿，积极推进高新区全域城市化等五个新型工业化综合配套专项改革；推动行政审批“两集中、两到位”职能归并；在各县区产业园区和服务业集聚区推行扁平化管理的体制机制创新；积极争取桓仁县成为全省扩权强县改革试点县。有效实施资金、土地要素保障。通过间接融资、发行城投债、引进股权投资基金、推进企业上市等方式，全年融资360亿元以上。通过推进新增建设用地、城乡建设用地增减挂钩、合理开发利用“三类用地”等方式，继续保障项目建设用地。坚持开放不止步，创新招商引资体制机制，进一步优化投资环境，加大“走出去、请进来”力度，开展境内外主题和专题招商60次，全年实际利用外资5.4亿美元，引进省外投资到位资金300亿元。

　　第二，继续实施项目年活动，全力推动重点项目建设实现新突破。按照招商引资增加项目、向上争取包装项目、结合信访稳定包扶项目三位一体的方式抓项目。继续坚持月通报、季调度，及时解决项目年活动中遇到的问题，努力形成“招商储备一批、前期工作一批、开工建设和续建一批、竣工投达产一批”的项目推进格局。今年要重点推进沈丹客专、本钢冷轧高强钢、石桥子互通立交改造、思山岭铁矿等项目建设的顺利实施，推动热电联产、田桓铁路、沈环高速公路本溪段、丹霍线千金岭隧道、观音阁输水工程等项目开工建设，积极推进本桓高速公路、桓仁抽水蓄能电站、桓仁支线机场和大台沟铁矿等项目的前期工作，努力推动1333个重点项目建设实现新突破。

　　第三，加快建设“钢都、药都、枫叶之都”，推进三大主导产业取得新进展。一是深化政企合作，推动钢都建设。继续创造空间和环境，全力支持本钢集团围绕“1+5”板块做精做强，推进新2号高炉改造等项目建成投产，支持本钢集团继续保持千亿元的销售收入和全国钢铁行业出口排名首位；进一步完善平山桥北、溪湖东风湖、南芬高新铸件等钢材深加工产业园区的总体规划、战略规划和产业规划，重点抓好天津银龙预应力钢绞线、恒通轧辊、金永新材等项目的开工建设和投产达产，全年引进钢材深加工项目50个，实现产值220亿元。二是继续弘扬药都精神，推动药都建设。落实“中国药都、健康之都，沈本新城、生态之城”的发展定位，加快建设健康产业集聚区。以8个产业园区为载体，加强对辉瑞、诺华和国药集团等知名医药企业的招商攻坚力度，全力促进成大、天士力等投产企业做大做强，全年引进生物医药、医药商业及其他新兴产业项目70个，新开工企业32户，新投产企业31户，药业及配套产业销售收入达到300亿元。加快沈阳药科大学主校区等4所大学建设。整合入驻高校资源，建设大学科技创业园。用好用足国家级高新区政策，积极建设药都创新园和创业园。以中央文化区日月岛核心区为重点建设沈本新城。年底前完成市民服务中心地下工程及部分地上主体工程，绿地中央文化广场15万平方米主体竣工。确保会展中心等工程竣工投入使用。蓝远名城项目一期竣工入住，二期封顶；公安业务技术大楼等项目开工建设。易辰药都商贸城全面开工建设，俊安金融广场完成主体工程。引入云服务计算中心项目，建立金融结算平台。启动创业、就业孵化基地商业一条街。规划战略性新兴产业园区，重点发展新一代信息技术等战略性新兴产业。三是坚持温泉旅游特色，推动枫叶之都建设。大力发展温泉旅游、乡村旅游、沟域旅游、文化旅游、庄园旅游，丰富枫叶之都内涵。加大水洞温泉旅游度假区、汤沟温泉谷项目的招商和建设力度，建设3个省内领先的温泉旅游小镇，培育3个10亿元以上的温泉旅游企业。实施“3个100”工程，本溪县新建20个特色旅游山庄，桓仁县新建20个冰酒酒庄，全市新建10个沟域旅游项目。争取本溪县、桓仁县获批成为辽东生态旅游示范基地。建设太子河新城至观音阁新城64公里旅游大道。打造平顶山文化品牌，促进环城森林旅游大发展。全年旅游总收入增长30%。

　　第四，统筹抓好农业、工业和服务业，构建三次产业协调发展新格局。大力发展现代农业。抓好“米袋子”、“菜篮子”工程。全市粮食作物播种面积70万亩以上，新建储粮仓5000个；继续实施好菜田建设规划。平地和林地山野菜种植面积达到6.5万亩，食用菌栽培达到3500万盘。在桓仁县建设2个万亩水稻高产创建示范区。完成小型农田水利工程100项，新增灌溉面积2.6万亩。加强重大动物疫病防控和畜产品安全工作。全面实施工业强市战略。以实施工业“五项工程”为抓手，长线抓投资，促进实体经济发展；短线抓提升，推进企业升级进位。规模以上工业企业总量达到637户；滚动实施400个投资千万元以上项目，完成工业固定资产投资400亿元；9个产业集群实现销售收入900亿元；完成国家、省下达的节能降耗和淘汰落后产能任务。积极发展非公有制经济，大力扶持中小企业和小微企业发展，鼓励和营造全民创业的氛围。加快发展服务业。全市房地产计划新开工800万平方米、竣工600万平方米；建筑业产值增长15%；重点推进太子河风光带及两侧开发等综合体项目。规划建设好8个服务业集聚区。实施“消费拉动、城区跨越和服务品牌”战略。发展“夜经济”，在重点城区各规划1个“夜经济”聚集区。发展物流经济，推动6个物流园区、1个物流中心建设取得实质性进展。发展会展经济，举办好药交会、枫叶节等大型会展活动。发展总部经济，重点吸引连锁零售贸易、物流企业的总部和结算中心。到年末，全市服务业要实现“三高两点”发展目标。

　　第五，突出特色发展县区经济，促进县区发展实现新跨越。县域经济要在“三化”建设上取得新突破。农业现代化突出“一县一业”，本溪县以建设“中国药都”优质原料生产基地为目标，积极发展中药材订单生产；桓仁县以建设“中国冰酒之都”为发展目标，壮大酿酒葡萄产业示范基地。工业化重点突出项目建设，本溪县人参铁加工和玻璃制品、桓仁县葡萄酒和包装印刷产业集群都要积极引进、培育投资超亿元的项目。城镇化要坚持县城和小城镇建设齐头并进，继续加快两县县城建设，推进基础设施和公共服务设施向乡镇覆盖。城区经济要在“两区”建设上取得新突破。工业园区建设要突出发展项目经济，率先破解土地、资金等要素瓶颈，同时加大特色引资力度。服务业集聚区要大力推进现代商业街、商业综合体等项目建设，推动各城区尽快形成一个主营业务收入达到50亿元的服务业集聚区。

　　第六，加强城市建设、环境保护和生态治理，打造宜居本溪新环境。坚持统筹主城改造与新城建设，加强以规划、市容为主的城市基础设施建设和城市管理。推进北地跨线桥建设，重点改造建设环山路、地工路等20条城市道路。实施城市绿化、美化、亮化、净化工程，城区栽植树木90万株，新增楼体亮化20栋。冲刺“创卫”工作，确保基本达到国家卫生城市标准。继续实施青山工程，完成超坡地还林2.25万亩，植树造林5.2万亩。巩固碧水工程成果，尽快形成污水处理能力，确保达标排放。全力推进“创模”工作，重点实施蓝天工程，落实49个大气治理项目，推进大气联防联控。组织实施50余项总量减排项目，确保完成年度减排任务。完成全域共享024区号任务，实现沈本通讯一体化。建设“宽带城市”，全面启动城市管理数字化平台建设。

　　第七，推进创新驱动和文化引领，增强城市竞争新动力。积极建设创新型城市。以高新区建设为引擎，充分发挥国家级高新区对高校、科研机构的辐射和聚集作用，健全创新机制，集聚创新资源，完善创新体系，增强自主创新能力，加快建设“创新本溪”。大力发展文化事业和文化产业。实施文化惠民工程，加强公共电子阅览室、乡镇综合文化站等公共文化基础设施建设，规划建设药都图书馆，对市艺术宫进行搬迁改造。建设本溪文艺家之家。推进市档案馆新馆建设。挖掘地域优势文化资源，促进文化与旅游、科技等深度融合，打造辽砚、剪纸、农民画基地等特色文化产业园区。做好庙后山遗址、本溪湖近代工业遗址群等文化遗产的保护和发掘工作。切实抓好人才队伍建设。重点加大高层次专业技术人才的选拔培育和急需紧缺人才的引进力度，要素和资源向人才集中，分配和激励向人才倾斜，在经济建设、社会管理、企业经营等领域形成高端人才集聚的局面。

　　第八，着力改善民生和发展社会事业，开创和谐社会建设新局面。今年要继续办好十大类、26件民生实事。一是围绕业有所就，全年完成实名制就业6.5万人；高校毕业生就业率达到95%以上；城镇登记失业率控制在4.1%以内。二是围绕多渠道增加城乡居民收入，全年争取惠农资金1亿元；帮助1万名农村低收入贫困人口脱贫；全市最低工资标准提高100元。三是围绕完善社会保障体系，将符合条件的城乡居民各类群体纳入相应社保制度覆盖范围，参保比例达到90%以上；资助城乡低保及边缘对象参加补充医疗保险。四是围绕住有所居，抓好国有工矿棚户区改造等安居工程项目建设，完成保障性安居工程8500套。五是围绕方便群众生活，沿道路两侧修建10个微广场；开发建设20个停车场；继续推进热源建设和管网改造，实施“暖房子”工程60万平方米；新建改造城市供水管网50公里；完成农村饮水工程63处。六是围绕学有所教，确保沈阳药科大学主校区等4所高校部分工程竣工；辽宁卫生职业技术学院开工建设；省实验学校本溪分校正式招生。七是围绕病有所医，全面落实基本公共卫生均等化项目；扩大国家基本药物制度覆盖面；确保辽宁中医药大学附属沈本医院投入使用。八是围绕丰富群众体育文化生活，建设溪湖区城中山公园等体育主题公园；继续承办CBA赛事。九是围绕老有所养，启动市级养老福利院建设；在各县区建立托养照料服务机构。十是围绕帮扶弱势群体，完成50个社区和500户贫困残疾人家庭无障碍改造；为100位农村单身贫困母亲援建住房。

　　同时，承办好篮球、公路自行车两个“十二运”赛事项目。抓好安全生产和信访稳定。继续推进平安本溪建设，以全国“十二运”安保为主线，深化公安信息化建设和城乡警务改革，不断提升驾驭社会治安动态管控能力和水平。加快建设本溪监狱新址工程。推进社会管理创新“九大体系”建设。抓好邮政市场监管。继续做好法制、人口计生、食品药品监管、人防、外事、侨务、对台、残联、民族、宗教、老龄、统计、档案、方志、气象、地震等工作。

　　各位代表、同志们，新的形势任务对新一届政府提出了更高要求。执政为民、重在落实，政府工作就是要狠抓落实。我们要坚定落实的信念和态度，心无旁骛地干、心底无私地干、心心相映地干、心甘情愿地干本溪人民所期盼的事业。要明确落实的方向和目标，围绕建设新本溪奋斗目标，真正做到明确目标、深入底层，深入底层、狠抓落实，狠抓落实、敢于担当，敢于担当、贵在争先。要完善落实的方法和路径，在认真落实中央八项规定、加快转变工作作风的同时，全力以赴争政策、抢项目、抓落实，时不我待拓空间、优环境、促发展。要形成落实的文化和团队，以欣赏的眼光、快乐的理念、包容的情怀、务实的举措，努力打造一个团结向上、开拓进取、勤政廉政的市政府团队。在狠抓落实的同时，我们要主动接受市人大的法律监督、市政协的民主监督和社会公众、新闻舆论监督，做好人大代表建议及政协提案办理工作。

　　各位代表、同志们，让我们共同努力，大干2013年，加快建设新本溪，为率先全面建成小康社会而努力奋斗！
